# Optimization of Innovation and Entrepreneurship Policy of Scientific and Technological Entrepreneur Talent Using Regional Competitive Advantage Theory

**DOI:** 10.3389/fpsyg.2021.726334

**Published:** 2021-12-14

**Authors:** Xiaoxuan Yu, Baogui Du

**Affiliations:** School of Humanity and Law, Northeastern University, Shenyang, China

**Keywords:** regional competitive advantage, scientific and technological talents, new entrepreneur, consciousness of innovation and entrepreneurship, educational psychology

## Abstract

Under the background of mass entrepreneurship and innovation, innovative entrepreneurship research is urgently needed for entrepreneurs. The aim was to explore the innovative entrepreneurship consciousness of new entrepreneurs. First, the regional competitive advantage theory is discussed. The research method of questionnaire survey is combined with statistical analysis to obtain relevant research data. Quantitative standards are used to measure the impact of regional advantages and policy support on entrepreneurs’ innovation and entrepreneurship. The policy content analysis and questionnaire survey are applied to discuss the impact of regional competitive advantage, educational psychology, innovation and entrepreneurship policy, and entrepreneurs’ innovation consciousness. Meantime, the policies related to entrepreneurs’ innovation and entrepreneurship are further explored. The results show that the proportions of young entrepreneurs suffering anxiety and depression are 29.4 and 27.5%, respectively, which are significantly higher than the national average. Besides, in the test, *F*=23.11, *p*<0.005, which indicates that all proposed hypotheses are valid. The results suggest that the overall mental health level of young new entrepreneurs is not high, and their consciousness of innovation and entrepreneurship needs to be strengthened. Under the wave of mass innovation and entrepreneurship, the research results can strengthen entrepreneurs’ innovative entrepreneurship consciousness and may have great theoretical and practical significance for improving and optimizing government innovation and entrepreneurship policies.

## Introduction

The current international economic situation is unstable, and China, which is deeply integrated into globalization, is also facing great downward pressure. The employment difficulty of college students caused by the year-by-year enrollment expansion of colleges and universities has become increasingly prominent, and the development of the times needs innovative and entrepreneurial talents. In 2014, Premier Li Keqiang puts forward mass entrepreneurship and innovation, hoping to promote employment by entrepreneurship and create a new engine for economic transformation and upgrading (Qu, 2021). Governments at all levels take active actions to promote policy implementation and policy support, especially, the intensive introduction of relevant supporting policies after the national innovation and entrepreneurship strategy is proposed. For example, the inland underdeveloped Jiangxi Province has issued a series of entrepreneurship support policies and optimizes the innovation and entrepreneurship environment, thereby stimulating the entrepreneurial passion of the masses. As the main force of innovation and entrepreneurship, young entrepreneurs have attracted the attention of all social spheres. Under the background of mass entrepreneurship and innovation, the government pays more and more attention to enterprise innovation and entrepreneurship, and the support of relevant policies is also increasing. The Fifth Plenary Session of the Nineteenth Central Committee of the Communist Party of China firmly grasps the main social contradictions in China, further puts forward the three-step strategy of scientific and technological innovation, and points out the direction for accelerating the construction of an innovative country.

The study aims to give better play to the advantages of regional resources, strengthen entrepreneurs’ awareness of innovation and entrepreneurship, and establish an advantageous and leading position in the process of enterprise competition. The theory of regional competitive advantage is combined with the statistical analysis data from the Questionnaire Survey (QS) and the policy research on the innovation and entrepreneurship of scientific and technological entrepreneur talents. Consequently, solutions are put forward based on quantitative standards to measure the influence of regional competitive advantages and policy support on entrepreneurs’ innovative entrepreneurship, and the influence of regional competitive advantage, educational psychology, and innovation and entrepreneurship policy on entrepreneurs’ innovation consciousness is explored through policy content analysis, principal component analysis, and linear regression. Significantly, the regional competitive advantage theory is innovatively applied instead of the traditional absolute advantage theory or the comparative advantage theory, and QS is combined to explore the influencing factors of innovative entrepreneurship of new entrepreneurs and policy optimization.

## Regional Competitive Advantage Theory and Innovative Entrepreneurship

### Literature Review

Domestic and international scholars have studied the entrepreneurial characteristics of new entrepreneurs. Specifically, Ayodotun et al. (2020) investigated the entrepreneurial intention and entrepreneurial characteristics of young and middle-aged people in economic development. The statistical analysis results showed that formal university education was closely related to the cultivation of psychological education, innovation consciousness, and the entrepreneurial intention of young people. Pankaj et al. (2020) analyzed the entrepreneurial behavior characteristics of tribal livestock farmers in the Kolhan region of Jharkhand. The results showed that the utilization rate of information resources and the level of psychological self-confidence of entrepreneurs also had a great impact on the entrepreneurial behavior of new entrepreneurs. Elena et al. (2020) discovered from the regional study from Romania that entrepreneurs’ perceived self-efficacy and problem-solving psychological self-confidence have different effects on their entrepreneurial success. Wu et al. (2018) studied the strategies of cultivating students’ practical ability of innovation and entrepreneurship by investigating students majoring in computer science in Guangzhou local undergraduate colleges and proposes to deepen the reform of innovation and entrepreneurship education in colleges and universities from multiple aspects. Ma et al. (2020) also proposed that universities should promote the integration of entrepreneurship education and professional education, change the concept of innovation and entrepreneurship education, and promote the reform of higher education. In short, the above research results indicate that there are still some deficiencies in the training policies of colleges and universities for young people’s innovative consciousness, of which, the biggest deficiency is the lack of inculcation of innovative consciousness of new entrepreneurs. Meanwhile, many young entrepreneurs do not realize that regional competitive advantages have a great impact on innovative entrepreneurship. With weak innovative consciousness, entrepreneurs present low entrepreneurship differentiation and cannot fully utilize the regional resources, so they are less competitive with their counterparts.

### From Absolute Advantage Theory to Comparative Advantage Theory

The traditional absolute advantage theory holds that if a country costs far less than other countries in specific product production, then it has the absolute advantage of that product, thus exporting that product. Each country has its favorable production conditions for specific products, and each country conducts international labor division according to its superior products and exchanges products.

The comparative advantage theory based on the theory of absolute advantage holds that it is very accidental that the two countries have the absolute advantage of different products. Each country does not have to produce products with absolute advantage but should concentrate on producing products with comparative advantages to export and import products with comparative disadvantages. This specialization can improve the productivity of labor.

### Theory of Regional Competitive Advantage

**Figure 1:**
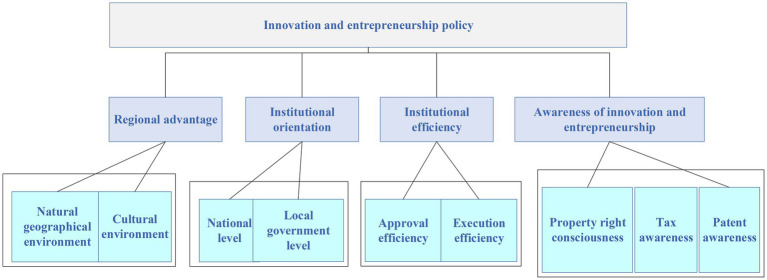
Four indicators of innovation and entrepreneurship policy affecting economic benefits.

Given the deficiency of comparative advantage theory, scholars creatively propose the theory of national competitive advantage based on the research of important trading economies for several years. The research suggests that in national and industrial competition, production factors are becoming less influential, while productivity has become the decisive factor, and productivity is determined by the competitive advantage. Although the theory of national competitive advantage is set at the national level, its analysis framework applies to regional and urban levels (Feng and Chen, 2020; Swazan and Das, 2021). Regional competitive advantage refers to the comprehensive competitiveness for regional product market share, which is determined by all schedulable resources within the region and regional difference factors, such as geographical environment, economic development, natural resources, and cultural environment in regional competition. The architecture of the impact of innovation and entrepreneurship policies on economic benefits is shown in Figure 1. The four indicators are the regional advantage, the institutional orientation, institutional efficiency, and consciousness of innovation and entrepreneurship, respectively. These indicators can also be subdivided into other factors. For example, regional advantages include natural geographical environment and cultural environment. Institutional orientation is divided into the national level and local government level. System efficiency includes system approval efficiency and system execution efficiency. The consciousness of innovation and entrepreneurship includes property rights consciousness, tax consciousness, and patent consciousness.

### Educational Psychology and Innovative Entrepreneurship

By integrating the guidance of educational psychology, innovation and entrepreneurship policy can improve the cultivation of innovation consciousness of new entrepreneurs. Educational psychology is the science to study the basic psychological laws, the psychological interaction between teachers and students, and the psychological phenomena in teaching and learning (Williams, 2021). Educational psychological analysis can obtain the mental health status of entrepreneurs to help them solve their mental health problems, cultivate their innovation consciousness, and help entrepreneurs develop innovative entrepreneurship utilizing local regional competitive advantages.

Innovative entrepreneurship is an entrepreneurial activity based on innovation, yet different from pure innovation or pure entrepreneurship. Innovation emphasizes pioneering and originality, while entrepreneurship emphasizes the behavior of obtaining benefits through practice. Therefore, in innovative entrepreneurship, innovation is the foundation of entrepreneurship, while entrepreneurship is the embodiment and extension of innovation (Deng et al., 2021; Duan et al., 2021). With the support of innovation and entrepreneurship policy, entrepreneurs can utilize the regional natural resources and geographical environment more conveniently, exert the regional competitive advantage, and carry out innovative entrepreneurship, thus improving regional economic development.

### Model Construction and QS Design

**Figure 2:**
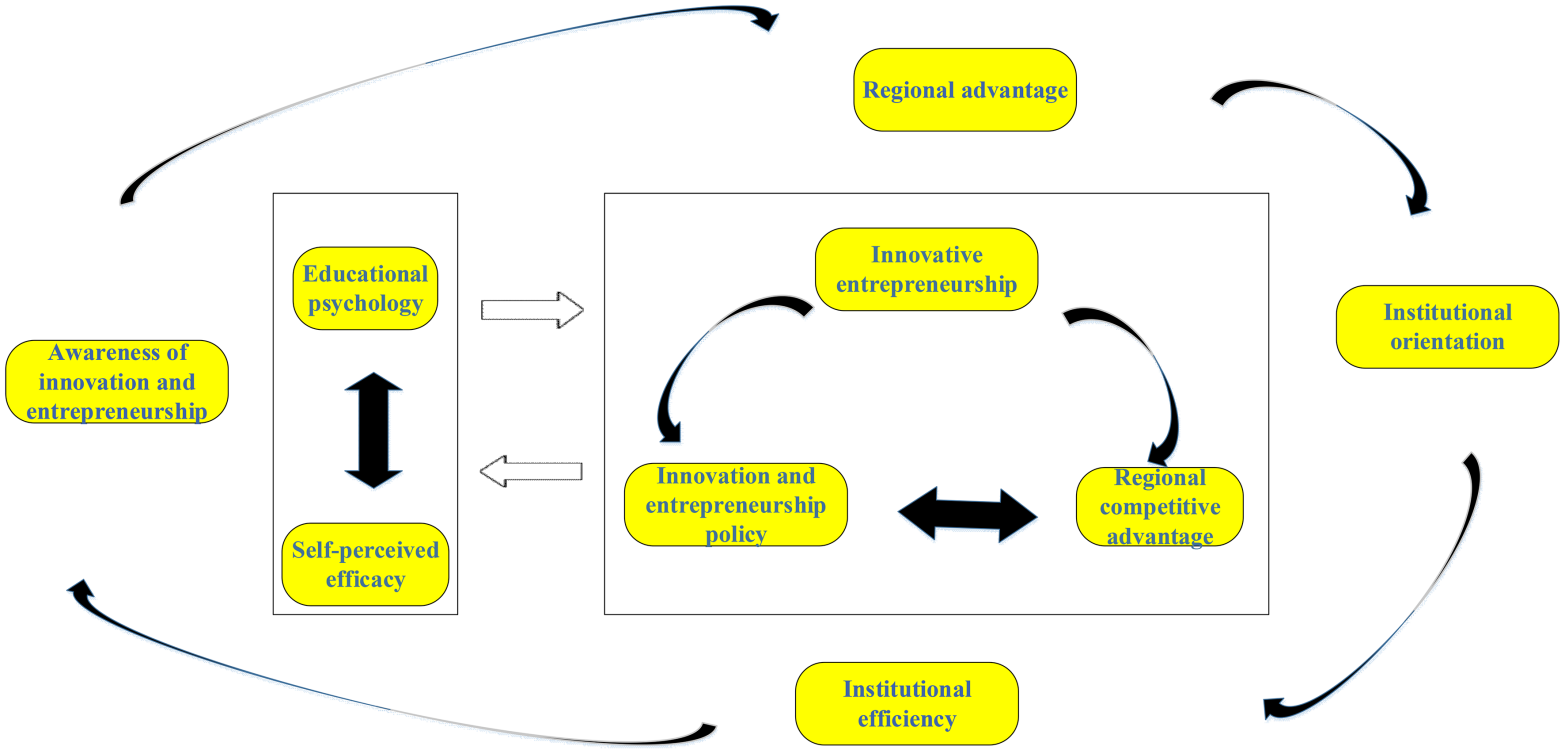
Technology roadmap.

According to the above analysis, the regional competitive advantage and the self-perceived efficacy of new entrepreneurs are closely related to innovative entrepreneurship. Self-perceived efficacy refers to an individual’s judgment of whether he can complete an activity at a particular level. In the regional competition, the greater the regional competitive advantage is, the more its attraction is to scientific and technological talents and new entrepreneurs, which can mobilize entrepreneurs’ innovation consciousness to start-up businesses, further developing local resources, form an industrial chain with comparative advantages, and accelerate economic development. The technology route map is shown in Figure 2. Figure 2 demonstrates that entrepreneurs’ self-perceived efficacy and educational psychology can interact with each other. The innovation of entrepreneurs, government innovation and entrepreneurship policies, and regional competitive advantages constitute the circular system of entrepreneurs’ innovation and entrepreneurship. These factors work together to influence the optimization of government innovation and entrepreneurship activities and policies.

**Figure 3:**
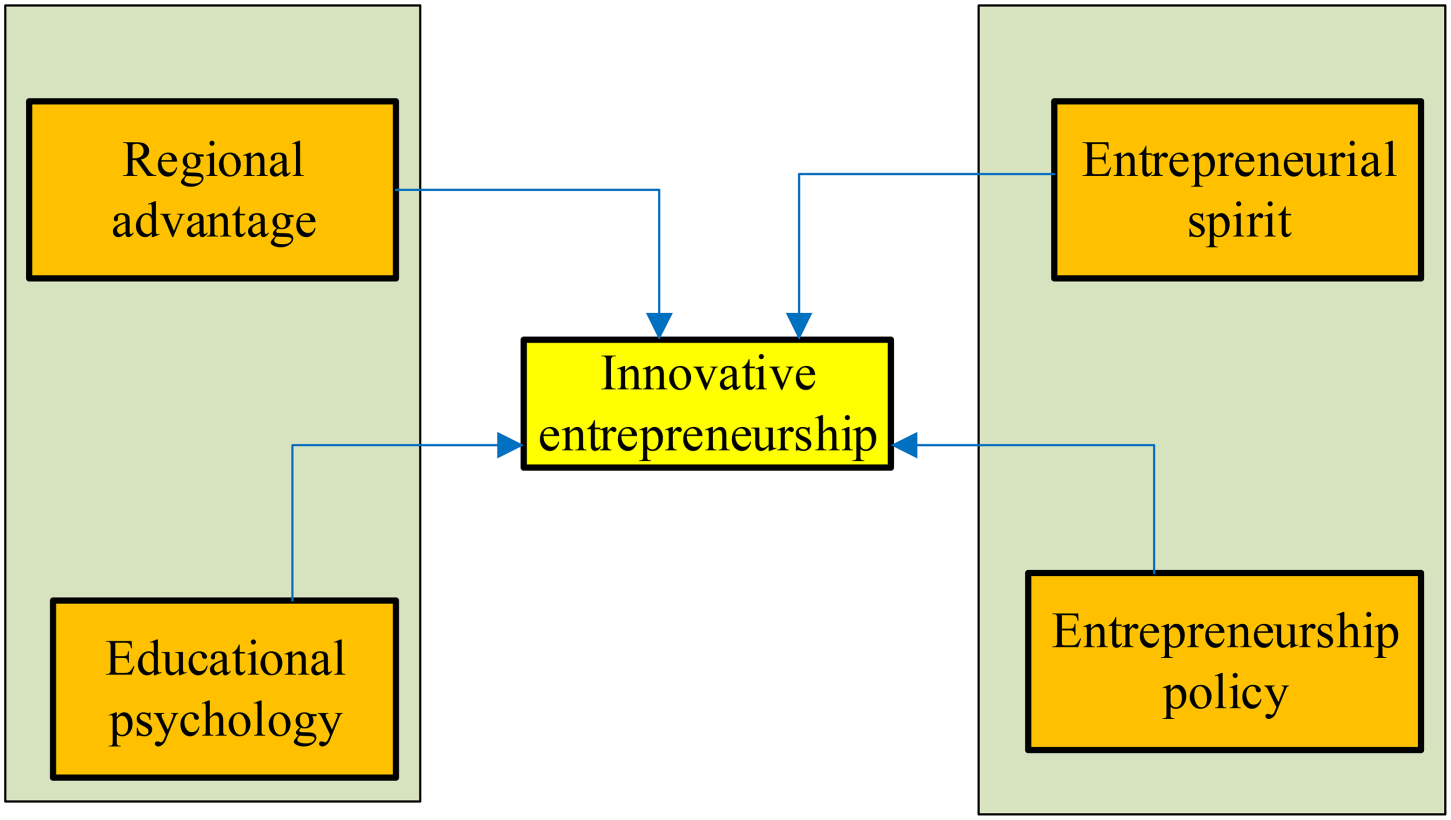
User intention research model.

Based on the regional competitive theory, a research model is proposed, as shown in Figure 3. Figure 3 shows that there are four important factors of entrepreneurs’ entrepreneurial behaviors, which are regional competitive advantage theory, educational psychology perception, entrepreneurial spirit of entrepreneurs, and government policies related to entrepreneurship.

### Questionnaire Survey (QS) Development and Design

The questionnaire on entrepreneurs’ innovation and entrepreneurship awareness is designed to collect the experimental data. The pre-test questionnaire relies on previously validated measurements and uses the Likert scale. After Professor Roger’s review and referring to Professor Roger’s relevant opinions (Roger et al., 2020), the following measures are proposed: entrepreneur’s innovation and entrepreneurship cognition and government innovation and entrepreneurship policy. All measurement results were verified by factor analysis. The measurement of variables used refers to the scale established previously. Correct the test level through translation and interpretation before formal investigation. Revise and adjust the scale with reference to the opinions of experts with relevant research experience and the intelligent service system, to improve the accuracy of the language and concept of the scale. The questionnaire is designed as shown in Table 1.

**Table 1:** A QS of entrepreneurs’ innovation consciousness based on regional competitive advantage.

Did you know about innovation and entrepreneurship training before starting a business?		
A. Do not know	B. Know a little	C. Know very well
How much do you know about the local entrepreneurship policy?		
A. Do not know	B. Know a little	C. Know very well
Do you know the local regional competitive advantages during entrepreneurship?		
A. Do not know	B. Know a little	C. Know very well
The self-efficacy of new entrepreneurs		
A. You can learn without being taught.	B. You can learn without a built-in assistant.	C. You can learn by being taught.

Concerning the validity and reliability of the QS, the QS is distributed among 160 entrepreneurs in Xi’an, including 102 men and 58 women, who all have started their own businesses. Of these, 55 of them have started their own business once, 70 of them have started their own business twice, and 35 have started their own business three times. Analysis of their understanding of regional competitive advantage theory and entrepreneurship policy is conducive to the optimization of policy research. For entrepreneurs with multiple start-up experiences, QSs are issued to collect their understanding of entrepreneurship policies in different entrepreneurship periods, and the correctness and validity of the results are tested through statistical analysis software. Cronbach coefficient is the most commonly in the reliability test. When the Cronbach coefficient is greater than 0.8, the scale has good reliability, and when the Cronbach coefficient is greater than 0.9, the scale has very ideal reliability. Thus, the Cronbach coefficient is used to study the reliability of the QS here. The results show that the value of Cronbach’s α coefficient is greater than 0.8, and the value of the Kaiser-Meyer-Olkin (KMO) is greater than 0.8, thus proving that the designed QS is reasonable and valid and can be distributed on a large scale. From September to December in 2020, a total of 300 QSs are distributed among new entrepreneurs in Shaanxi Province, 255 are recovered, and 241 are effective, with an effective rate of 80.3%. Significantly, Cronbach’s α coefficient can test the reliability of the questionnaire. The larger the Cronbach coefficient, the higher the internal consistency of the QS. If the Cronbach coefficient is less than 0.65, the reliability of the QS is low. If Cronbach’s α coefficient is greater than 0.65 and less than 0.7, the QS is acceptable. If the Cronbach’s α coefficient is greater than 0.8, the reliability of the QS is very high. Thus, the designed QS has high reliability (Norsa’adah et al., 2020).

## Results Analysis

### Analysis of QS Results

**Figure 4:**
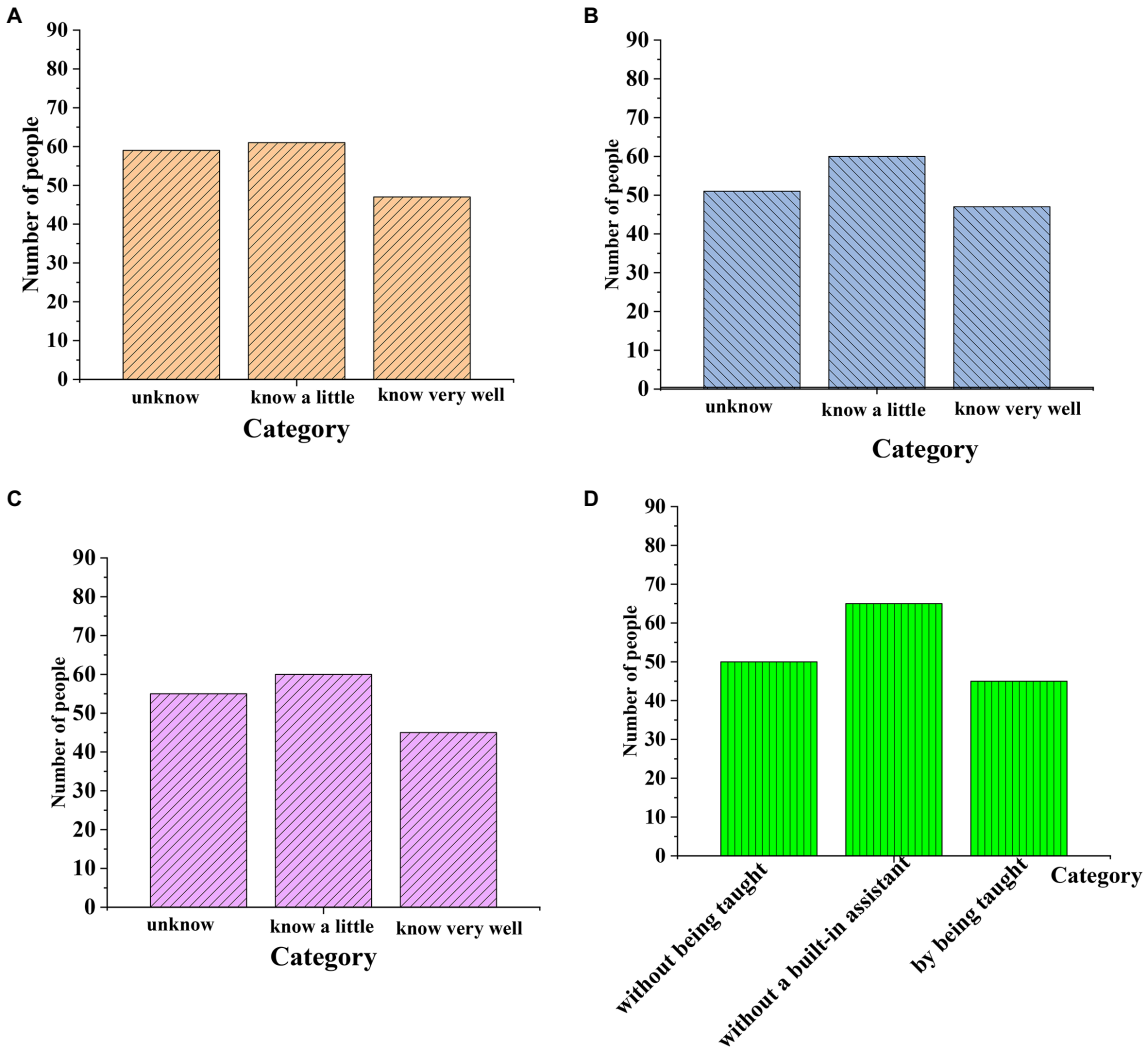
QS analysis results: **(A)** shows the degree of understanding of entrepreneurial training, **(B)** S illustrates the degree of understanding of entrepreneurial policies, **(C)** indicates the understanding of regional advantages, and **(D)** reveals the self-efficacy of entrepreneurs.

The results of the QS are shown in Figure 4. Figure 4 suggests that the peak of the concentration curve is located in the center, that is, the position of the mean, and the curve at the mean is the highest. The symmetry curve takes the mean as the center and gradually decreases evenly to both sides, and the area under the curve has a certain distribution law, so the image data conform to the data of normal distribution. Figure 4 shows that the sample data conform to the normal analysis, and the number of samples is sufficient, which can be used for the establishment and fitting analysis of the structural equation model. The QS results indicate that the proportion of male respondents is high. Additionally, the respondents’ overall understanding of entrepreneurship policy and regional competitive advantage theory is not high, and they have not made full use of self-perceived efficacy and entrepreneurial advantage during entrepreneurship. Overall, however, the sample is representative. The reliability and validity of the data prove that the QS is feasible, meets the premise of structural equation model validation, and measures up to the standards of data analysis and empirical test.

### Reliability and Validity of the QS

**Figure 5:**
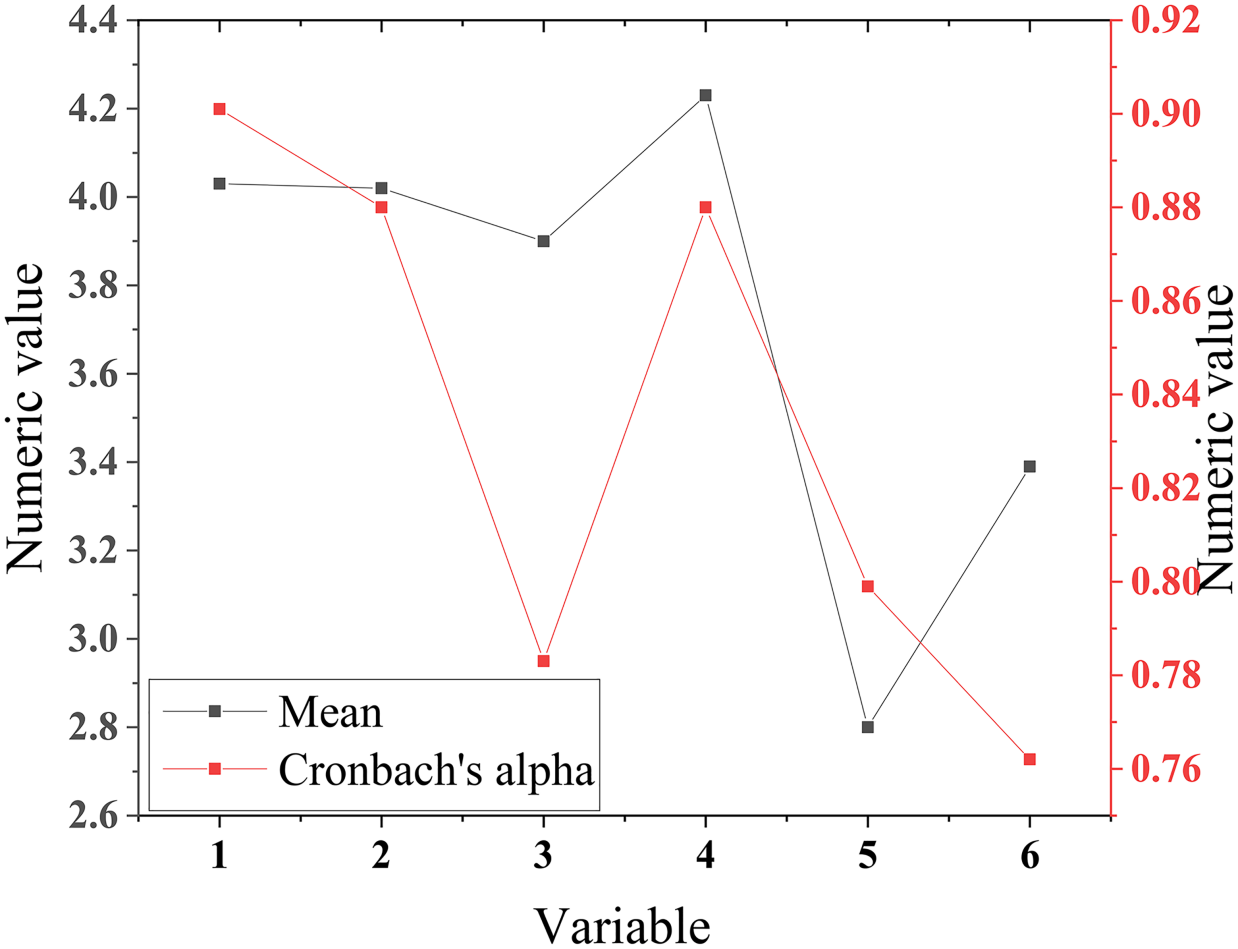
Reliability test results of the QS.

The reliability of each measurement item in the QS is tested. Cronbach’s α coefficient and the signal value of the reliability test are shown in Figure 5.

Figure 5 implies that the designed QS is more reasonable and reliable, which meets the reliability test standard. Cronbach’s α coefficient of the QS is 0.922, and Sig (Significance indicator) is 0.000. The test results show that the internal consistency, reliability, and repeatability of the internal measurement items are good, and the QS is reliable.

**Figure 6:**
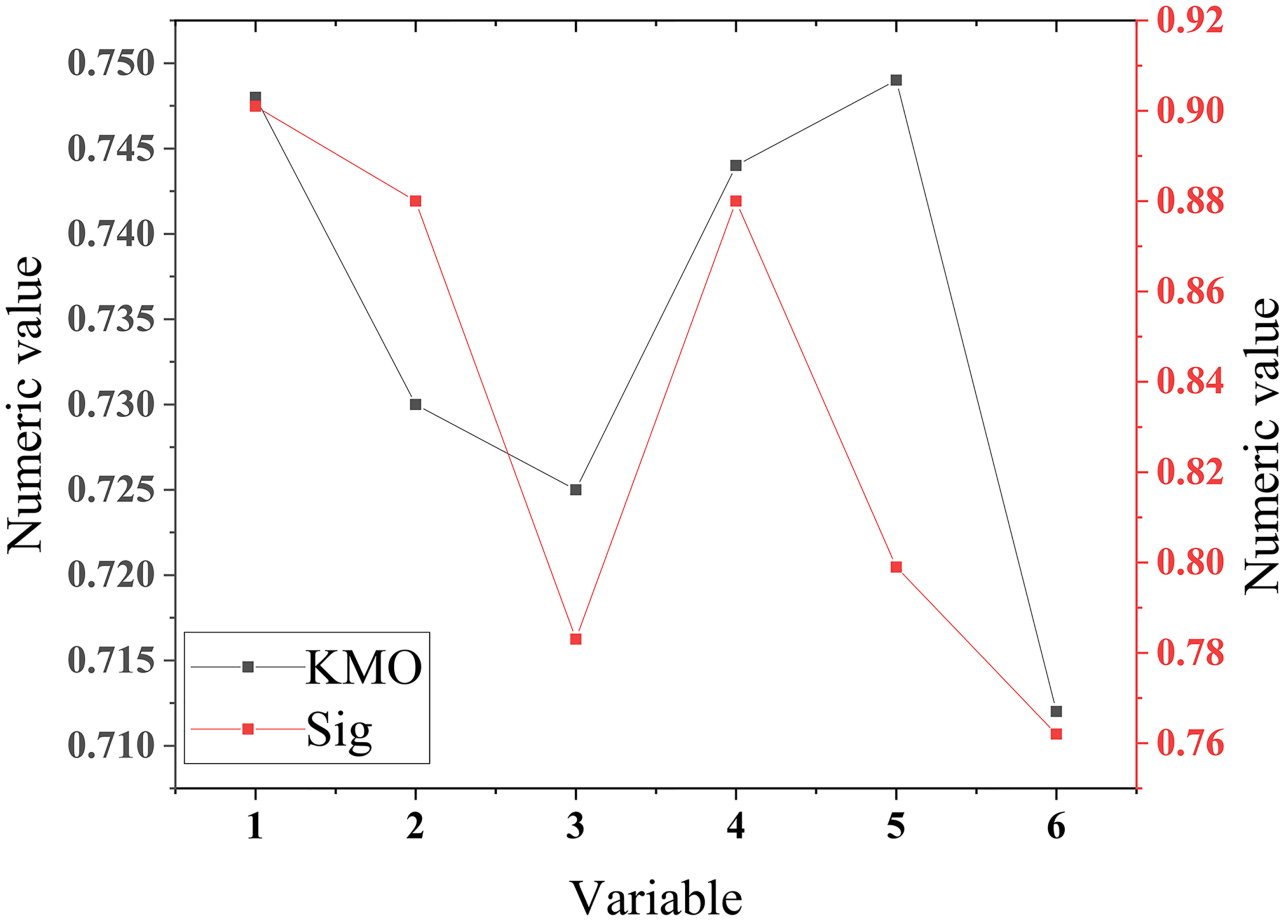
Kaiser-Meyer-Olkin test of the QS.

KMO test statistics can verify whether the samples, and QS can be verified through factor analysis. The KMO values of each variable in the QS and the statistical results of the Bartlett sphere test are shown in Figure 6.

Figure 6 illustrates that the KMO values of perceived ease of use, perceived usefulness, self-efficacy, and perceived external control are 0.748, 0.730, 0.725, and 0.744, respectively. Meanwhile, the chi-square of perceived ease of use, chi-square of perceived usefulness, chi-square of self-efficacy, and chi-square of perceived external control are 536.213, 462.512, 289.112, and 483.112, respectively. The KMO test values of all variables in the QS are greater than 0.7, all Sig values are 0, and the chi-square values of the spherical test are greater than 200. This shows that the variables in the QS are suitable for factor analysis (Lu et al., 2020). The analysis results of the whole QS indicate that the QS has passed the reliability and validity test, and the overall effect meets the expectations.

### QS Results of the Mental Health of New Entrepreneurs

**Figure 7:**
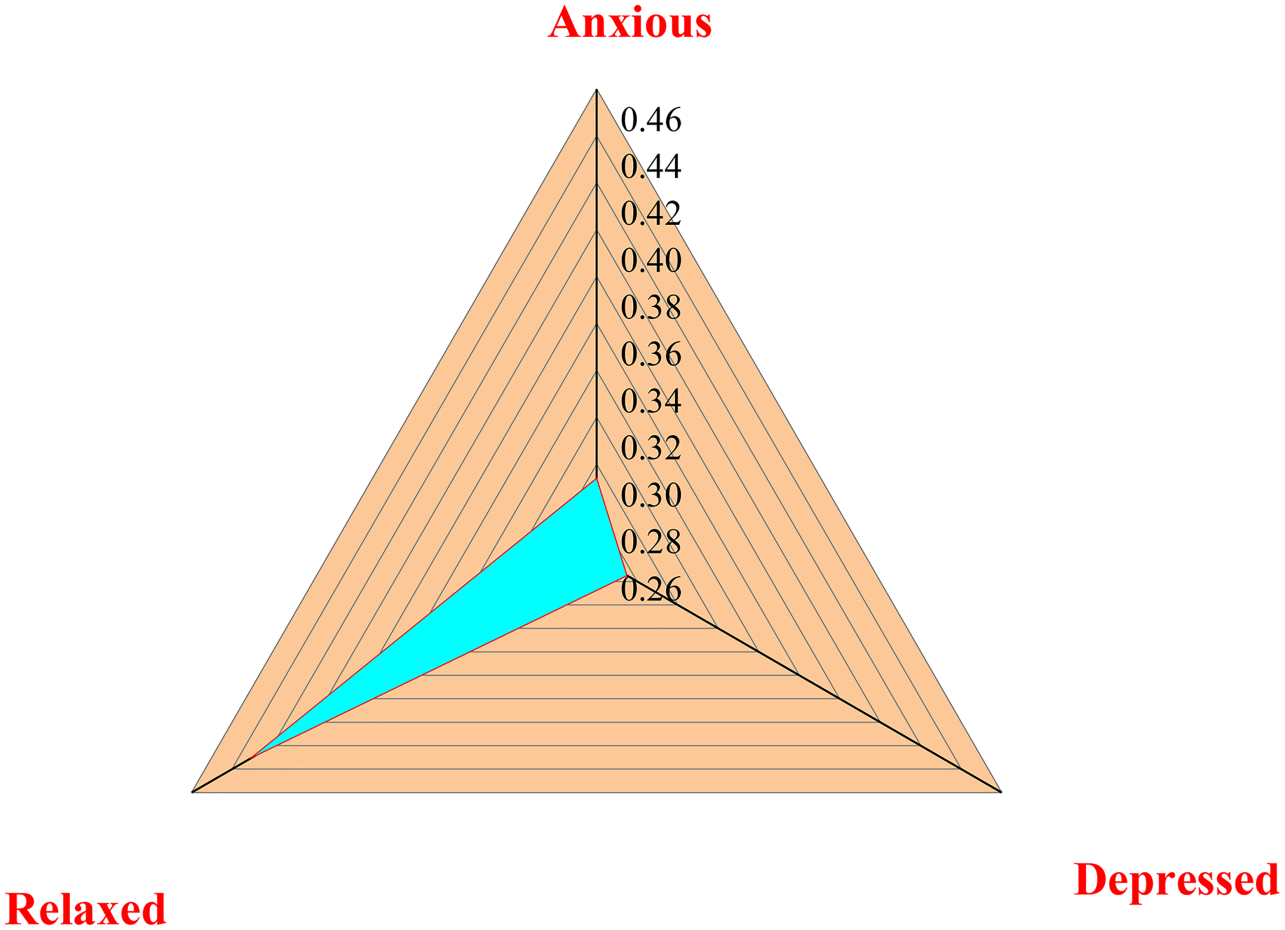
Analysis of mental health of young entrepreneurs.

The QS results suggest that the mental health status of young entrepreneurs is not optimistic. Due to the professional characteristics and competitive environment, young entrepreneurs’ pressure mainly comes from work and social responsibilities, and their mental health problems are serious (Wang, 2020). The QS results of entrepreneurs’ mental health are illustrated in Figure 7.

Figure 7 implies that 29.4% of the surveyed new entrepreneurs have felt anxiety, 27.5% of the respondents have felt depression, and only 43.1% of the subjects have not felt anxiety or depression. Thus, the solutions are to carry out mental health education for new entrepreneurs using the psychological education theory, solve their mental health problems of entrepreneurs, and cultivate their consciousness of scientific and technological innovation with innovation and entrepreneurship policies.

## Discussion and Suggestions

Based on the above analysis, following points can be summarized. To make entrepreneurship and innovation popular and fruitful, rather than a flash in the pan, the reform should be further deepened and institutional barriers should be broken to liberate and lead the innovation and entrepreneurship wave. Meanwhile, innovation and entrepreneurship education should be strengthened, resources and policies should be integrated and implemented. Su et al. (2021) investigated the influencing factors of Chinese college students’ entrepreneurial intention, used the perspective theory of planned behavior combined with perceived university support to expand the framework theory of planned behavior, and explained the impact of this support on students’ entrepreneurial intention. The results showed that perceived university support significantly affected students’ attitude toward entrepreneurship, which showed that universities played a key role in cultivating students’ entrepreneurship. Further, the institutions and mechanisms should be restructured to encourage innovation and development. At the same time, the reform of the administrative system should be deepened to abolish and decentralize the administrative examination and approval items, clean up the so-called non-administrative licensing, promote the negative list system of market access, and lighten the burden of market subjects. Intellectual property rights will be protected under the law, as well as other legal property rights. A new economic engine should be built based on the promotion of scientific and technological innovation through institutional reformation. Liu et al. (2021) studied the cultivation of college students’ professional innovation ability through the college students’ innovation and entrepreneurship training program. Taking the college students’ innovation and entrepreneurship training program as an example, combined with the author’s own participation experience, they discussed the impact of the college students’ innovation and entrepreneurship competition on the cultivation of college students’ professional innovation ability. The emerging industries should be further developed, and middle and high-end industrial restructuration should be promoted. Most importantly, innovation projects should be implemented, such as special projects or government procurement for the start-up enterprises to stimulate the start-up enterprises to enter the market. For key entrepreneurial groups, such as overseas returnees and college graduates, self-employment support projects can be set up funded by the national or local governments. In terms of supporting youth entrepreneurship, international experiences can be learned to set up a special youth entrepreneurship plan, coordinate various departments, and form a policy joint force to support innovative entrepreneurship. The main contribution is to learn from international experience to support the implementation of youth entrepreneurship policy, analyze entrepreneurship policy by using regional competitive advantage theory, set up special youth entrepreneurship plan, and coordinate various departments, which can form a policy joint force to support innovation and entrepreneurship.

Moreover, various forms of innovation and entrepreneurship curriculums should be brought into higher education. Colleges and universities should improve the entrepreneurship methods for college students, encourage students to learn through enterprise practice, and ensure the combination of entrepreneurship curriculum and practice (Duan et al., 2021; Li et al., 2021). Colleges and universities should develop entrepreneurship training through cooperation with training institutions, build the entrepreneurship incubation base, further improve the entrepreneurship training service system, and provide targeted training and entrepreneurship services for college students. Locally, the college students’ entrepreneurship guidance plan should be implemented according to the actual situation, and the national strategy to promote the Internet + comprehensive development should be formulated, thereby supporting the innovative entrepreneurship of college students. Significantly, strategies and guidelines should be formulated from top to bottom to promote the healthy development of the Internet + and the integration of the Internet and the traditional industries in various forms, such as Internet + finance, Internet + transportation, and Internet + medical in terms of technology, standards, and policies.

Further, traditional industries should be transformed through process innovation, led by product innovation, and based on mechanism research to improve the international market competitiveness (Jena, 2020). Firstly, backward technologies and excess capacity should be eliminated and digested timely, while the merger and reorganization of overcapacity and high-pollution industries should be accelerated. Meanwhile, the overcapacity industries should promote equivalent capacity replacement with new technologies, and new capacity should be strictly controlled. Secondly, advanced technologies in traditional industries should be fully utilized to develop high-end manufacturing and strategic emerging industries. Besides, more efforts should be exerted on research and development of advanced materials, product innovation, and new models, and basic research should be reinforced to improve the international market competitiveness. Thirdly, fiscal and tax reform should be implemented, new measures should be launched to support Small and Medium-sized Enterprises, financial reform should be deepened, and the marketization of interest rates and exchange rates should be promoted (Liu and Hong, 2019). Especially, the development of small and medium-sized financial institutions, such as private banks, should be accelerated, and specifically, the capital market should be expanded, price reform should be promoted, government pricing should be minimized, while price liberalization should be maximized. At the same time, the government’s role should be fully exerted in the construction of a soft environment and market supervision to create an international, market-oriented, and legal environment for entrepreneurship and innovation and provide high-quality and efficient public services for all market players.

Lastly, financial support and credit policies should be fully implemented in mass entrepreneurship to guide financial resources to public goods and services, promote the innovation of financial products and services, expand the scale of direct financing, and build diversified financing channels for public goods and services (Peng et al., 2020). First, in the field of science and technology, financial institutions should be guided to increase credit investment in science and technology enterprises, especially, science and technology MSE, deepen the combination of science and technology and finance, strengthen policy coordination in science and technology, finance, and taxation, and finance, and jointly promote the development of science and technology finance (Galindo-Martín et al., 2020). Second, the development of Internet finance should be standardized and regulated to prevent and resolve financial risks. Meanwhile, it is necessary to further improve and perfect the financial services in the fields of education and culture, explore suitable mortgage and pledge methods, develop education and cultural consumer credit products, provide more convenient payment and settlement services, and improve the convenience of trade investment and financing in education and cultural industries (Mok et al., 2020).

## Conclusion

Here, the QS data are comprehensively used and deeply mined. As a result, patterns contained in the data are discovered to verify the role of regional competitive advantages for the innovative entrepreneurship of new entrepreneurs. The results show that the regional competitive advantages have a great influence on the innovative entrepreneurship of new entrepreneurs, but the mental health level of the new entrepreneurs is generally low: 29.4% of the surveyed entrepreneurs have felt anxiety, and 27.5% of the respondents have felt depression. This demonstrates that the psychological quality of scientific and technological entrepreneur talents is generally weak, and their consciousness of innovation is insufficient, so they cannot make good use of the theory of regional competitive advantage to solve the problem of innovative entrepreneurship. Here are the deficiencies. The QS only statistically analyzed the data of just over 160 new entrepreneurs in Xi’an City, so the research scale is relatively small, and in the subsequent research, the sample scale will be further enlarged.

## Data Availability Statement

The raw data supporting the conclusions of this article will be made available by the authors, without undue reservation.

## Ethics Statement

The studies involving human participants were reviewed and approved by Northeastern University Ethics Committee. The patients/participants provided their written informed consent to participate in this study. Written informed consent was obtained from the individual(s) for the publication of any potentially identifiable images or data included in this article.

## Author Contributions

All authors listed have made a substantial, direct and intellectual contribution to the work, and approved it for publication.

## Conflict of Interest

The authors declare that the research was conducted in the absence of any commercial or financial relationships that could be construed as a potential conflict of interest.

## Publisher’s Note

All claims expressed in this article are solely those of the authors and do not necessarily represent those of their affiliated organizations, or those of the publisher, the editors and the reviewers. Any product that may be evaluated in this article, or claim that may be made by its manufacturer, is not guaranteed or endorsed by the publisher.
